# 3D Bioprinting Strategies in Autoimmune Disease Models

**DOI:** 10.3390/ijms27010343

**Published:** 2025-12-29

**Authors:** Natalia Wiewiórska-Krata, Bartosz Foroncewicz, Radosław Zagożdżon, Krzysztof Mucha

**Affiliations:** 1 Laboratory of Cellular and Genetic Therapies, Center for Preclinical Research, Medical University of Warsaw, 02-097 Warsaw, Poland; 2 ProMix Center (ProteogenOmix in Medicine), Department of Clinical Immunology, Medical University of Warsaw, 02-006 Warsaw, Poland; 3 Department of Transplantology, Immunology, Nephrology and Internal Diseases, Medical University of Warsaw, 02-006 Warsaw, Poland; 4 Institute of Biochemistry and Biophysics, Polish Academy of Sciences, 02-106 Warsaw, Poland

**Keywords:** bioprinting, autoimmune diseases, disease modelling, treatment response, precision medicine

## Abstract

Three-dimensional (3D) bioprinting is a rapidly evolving technology that uses complementary biomaterials to emulate native extracellular matrices, enabling the generation of finely patterned, multicellular tissue architectures. Autoimmune diseases (AD), which are characterized by chronic, often organ-specific, immune response, are ideally suited to these in vitro models. This review summarizes the current state of 3D bioprinting for modelling AD, focusing on rheumatoid arthritis (RA), type 1 diabetes (T1D) and inflammatory bowel disease (IBD), as well as applications to systemic lupus erythematosus (SLE), neuroinflammatory conditions such as multiple sclerosis (MS) and other AD. Bioprinting modalities, advances in immune competent bioinks, strategies for vascularization and approaches to the hybridization of printed tissues with organoids and organ-on-chip systems are reviewed. From a clinical perspective, this review focuses on applications with translational potential, including immune-competent models derived from patients for biomarker discovery, drug screening and treatment response prediction. The key challenges, notably the reconstitution of full immune complexity, stable and perfusable vasculature, and maintenance of long-term viability and function are highlighted. Finally, future directions are defined to enhance the clinical utility and impact of 3D bioprinting across preclinical development and precision medicine.

## 1. Introduction

Autoimmune diseases (AD) affect approximately 8–10% of the global population and are associated with significant morbidity [1,2]. The pathogenesis of these diseases is driven by chronic inflammation, exaggerated/misguided immune response and tissue remodeling [2]. From the clinical perspective, standard treatment relies on the use of immunosuppressive agents, monoclonal antibodies [3,4] or immunoglobulin replacement therapies [5]. However, the broad range of side effects and reduced quality of life associated with these treatments warrants the search for more tailored and efficient strategies [6,7].

**Figure 1:**
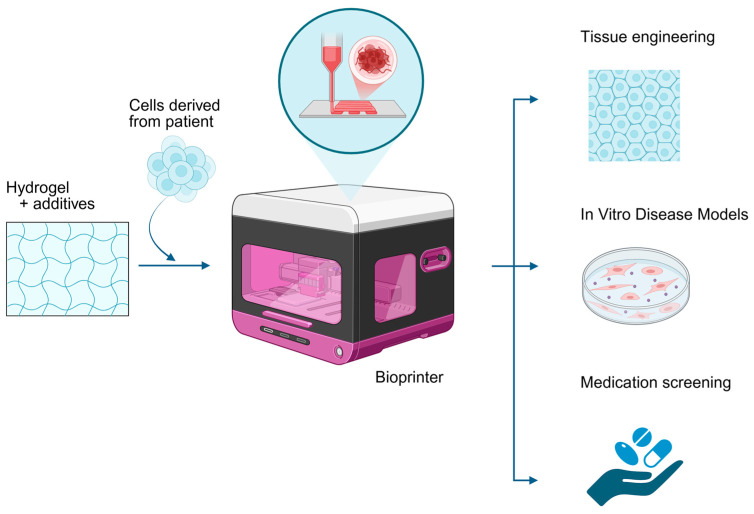
The 3D bioprinting procedure and potential applications.

Recent advances in three-dimensional (3D) bioprinting have enabled the fabrication of biological systems containing living cells, which have significant implications in medicine [8]. These technologies offer valuable tools for AD treatment studies [9]. Traditional two-dimensional (2D) cultures are limited in their ability to replicate cell-to-cell interactions, matrix architecture and immune infiltration. In contrast, 3D cultures more accurately model native tissue physiology, disease pathology and treatment responses [10]. In addition, 3D bioprinting allows for the precise arrangement of multiple cell types, extracellular matrix (ECM) components and bioactive molecules, thereby closely mimicking native tissue architecture [11]. In AD research, this technology enables manufacturing of tissue models for controlled investigation of immune-tissue interactions [12]. Such models promise more predictive platforms for tissue engineering, preclinical drug testing (Figure 1), personalized medicine approaches and studies on disease pathogenesis [8,13].

## 2. 3D Bioprinting for Autoimmune Disease Modeling

### 2.1. Bioprinting Techniques

Multiple bioprinting modalities are employed to construct AD models. Among them extrusion-based bioprinting, which employs pneumatic or mechanical pressure to deposit continuous filaments of cell-laden hydrogels, is the most widely adopted [8]. Its compatibility with viscous, high-cell density bioinks makes it suitable for fabricating tissue constructs that require substantial structural integrity [14]. However, the resolution of this technique remains limited, as the smallest achievable feature size typically ranges between 200 and 1000 μm, making it difficult to replicate the fine structural complexity of native biological tissues [15]. Moreover, optimizing printability often comes at the expense of cell viability, highlighting the inherent trade-off between structural precision and biological integrity [16].

Inkjet bioprinting utilizes thermal or piezoelectric pulses to eject droplets of low-viscosity bioinks, allowing for high-resolution deposition of biomolecules. However, this technique is constrained by shear stress and nozzle clogging at higher cell densities [17], limiting its application in the engineering of mechanically robust tissues [18].

Laser-assisted bioprinting which provides even higher spatial resolution, using laser pulses to transfer cell-containing droplets onto substrates, preserves high cell viability and has been used to generate microvascular structures, essential for modeling immune infiltration [19]. However, it remains limited by high operational costs, complex calibration requirements and low scalability, particularly when fabricating large or clinically relevant tissue constructs [20].

Recent innovations in 3D-embedded bioprinting, including Freeform Reversible Em-bedding of Suspended Hydrogels (FRESH) and Suspended Layer Additive Manufacturing (SLAM), have expanded the capacity to print soft, low-viscosity bioinks with high spatial precision. The FRESH technique, first described by Hinton et al. [21], enables high-fidelity fabrication of soft, natural biomaterials, including collagen and fibrin, within a gelatin support bath, maintaining excellent cell viability and geometric accuracy. This approach allows the generation of complex, anatomically relevant constructs, such as vascular and cardiac models, that are difficult to achieve with conventional extrusion-based methods. However, FRESH bioprinting remains limited by the low mechanical stability of the printed constructs and the need for bioink-specific optimization of the support bath. Additionally, scaling up to clinically relevant tissue volumes is limited by prolonged printing times and challenges associated with clean removal of the support material [22]. The SLAM approach, according to Cooke et al. [23], discusses the structuring of hydrogels to improve their functionality in biomedical applications. The authors highlight how controlled shear processing and self-assembly principles enable the formation of injectable ‘fluid gels’, and printable materials well-suited for drug delivery, tissue regeneration and bioprinting. However, despite their versatility and reliance on regulator-approved polymers, these hydrogels remain limited by mechanical weakness, sterilization challenges and difficulties in large-scale manufacturing; these challenges must be addressed to achieve clinical translation [23]. Both the FRESH and the SLAM technique offer significant advantages for fabricating complex immune and vascularized constructs, such as lymphoid and pancreatic microtissues, which are challenging to reproduce using conventional extrusion-based bioprinting methods [21,23,24].

Beyond scaffold-based strategies, scaffold-free bioprinting has gained significant popularity as an innovative approach for engineering tissue-like constructs. These constructs rely on cellular self-organization rather than external matrices. In systems such as spheroid fusion, tissue strands, and cell-sheet stacking, cells autonomously deposit ECM and establish native intercellular signaling networks [25]. This self-directed assembly more closely mirrors natural tissue morphogenesis, offering particular advantages for studying immune-tissue interactions in autoimmune diseases. Recent studies have demonstrated that scaffold-free constructs can successfully reproduce lymphoid microenvironments and vascularized organoids, enabling the investigation of immune cell infiltration, cytokine exchange and inflammatory cross-talk under physiologically relevant conditions [26,27]. In the context of autoimmune disease modeling, these self-assembled systems hold great promise for improving biological fidelity [28]. By eliminating artificial scaffolds, they reduce diffusion barriers and foreign material effects, thereby more accurately recapitulating biological processes [25]. These processes, include, among others, T cell infiltration in rheumatoid synovium or β-cell–immune interactions in type 1 diabetes [28,29]. The major bioprinting strategies for AD modelling are summarized in Table 1.

### 2.2. Bioink

**Figure 2:**
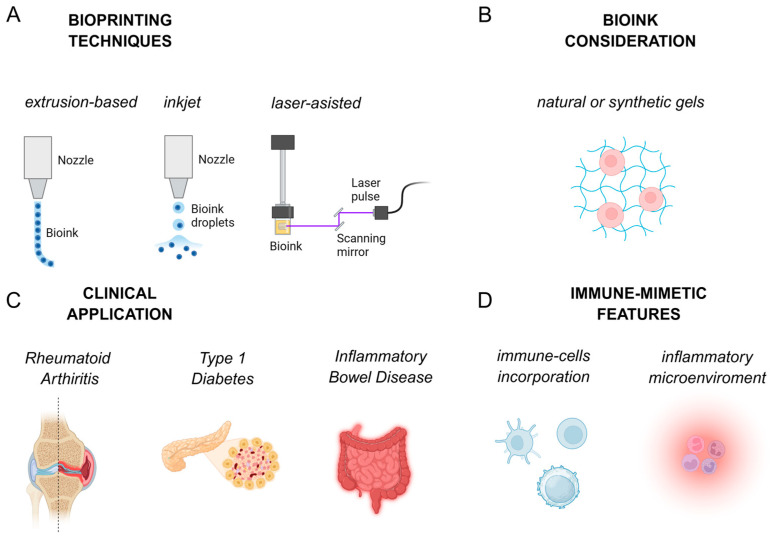
Overview of bioprinting approaches and applications in AD modeling. (**A**) Schematic representation of three principal bioprinting strategies. (**B**) Bioink consideration—natural or synthetic gels. (**C**) Representative disease models demonstrating the translational potential of 3D bioprinting in AD. (**D**) Incorporation of immune cells and simulation of inflammatory microenvironments as a strategy for immune-mimetic applications.

The design of bioinks is critical for manipulating biological and biochemical environments to generate complex disease models [17]. The natural hydrogels, such as collagen, gelatin, hyaluronic acid and alginate, provide biocompatibility and cell-adhesive motifs, frequently replicating the native ECM structure [35]. Although natural hydrogels are valued for their excellent biocompatibility and inherent cell-adhesive properties, they also come with important limitations. Their composition can vary from batch to batch, and their mechanical strength and degradation rate are often difficult to control, which can compromise reproducibility and limit the stability of long-term cultures [35,36]. In addition, their limited thermal and rheological stability makes achieving precise printing challenging, especially when fabricating large or mechanically demanding constructs [37]. To address these limitations, synthetic hydrogels, including polyethylene glycol and Pluronic, are preferable, offering tunable, mechanical properties and customizable chemical functionality to better replicate inflammatory microenvironments [36,38]. The structural and compositional variability of gels influence their biocompatibility, mechanical stability and immunogenicity [39]. In AD models, the inclusion of cytokines or ECM derived from diseased tissues further enhances pathophysiological relevance [40]. The incorporation of immune cells and simulation of inflammatory microenvironments within printed constructs enables the reproduction of disease-specific immune responses and tissue remodeling. The overview of bioprinting approaches and applications in AD modeling is presented in Figure 2.

### 2.3. Application of 3D Bioprinting in AD Models

#### 2.3.1. Rheumatoid Arthritis (RA)

Rheumatoid arthritis (RA) is a chronic, systemic AD characterized by persistent synovial inflammation, symmetric polyarthritis and progressive joint destruction [41,42,43]. It primarily affects synovial joints and periarticular soft tissues, leading to pain and swelling, and ultimately to irreversible deformity [43]. The global incidence and prevalence of RA vary substantially, with higher rates observed in Europe and North America compared with Southeast Asia [44]. Over the past three decades, the prevalence and incidence of RA have increased by approximately 7.4% and 8.2%, respectively [45].

The pathogenesis of RA arises from a complex interplay between innate and adaptive immune responses that target self-antigens within the synovium. The earliest immunological events include the production of ACPAs and rheumatoid factor. These antibodies form immune complexes that activate complement and resident immune cells [46,47]. Local antibody production is promoted by IL-21 and CXCL13, secreted by follicular helper CD4^+^ T cells, upon recognizing citrullinated peptides presented by antigen-presenting cells [48]. Concurrently, CD8^+^ cytotoxic T cells and Th17 cells secrete proinflammatory cytokines, such as IFN-γ, IL-17 and GM-CSF, which further amplify inflammation [49,50,51]. Activated macrophages and neutrophils release TNF, IL-1β and IL-6, which stimulate fibroblast-like synoviocytes (FLS) to proliferate, migrate and produce matrix-degrading enzymes [49,51]. FLS also releases TGF-β and IL-13, promoting fibrotic remodeling and synovial hyperplasia. Monocytes differentiate into osteoclasts, contributing to bone erosion and joint destruction [52]. Meanwhile, a specialized subset of tissue-resident macrophages forms an immune-regulatory tight junction barrier, that constrains excessive inflammation, highlighting an intrinsic immunoregulatory axis within the synovial microenvironment [53]. Dysregulation of these homeostatic mechanisms drives the transition from localized synovitis to systemic autoimmunity, underscoring the complex immunopathology of RA [41,54,55].

The advancement of 3D bioprinting has facilitated the development of advanced platforms to RA pathogenesis, therapeutic screening and joint tissue regeneration under controlled, physiologically relevant conditions [31]. Conventional in vitro models and animal systems are limited in their ability to replicate the cellular complexity, mechanical microenvironment and chronic immune activation characteristic of human synovitis [41,42,44]. Bioprinting overcomes these limitations by enabling spatial organization of synovial fibroblasts, chondrocytes, immune cells and vascular structures within extracellular-matrix-mimicking hydrogels that reproduce the inflammatory and mechanical cues present in the arthritic joint [56]. Bioprinting enables the fabrication of synovium-like constructs by embedding FLS, macrophages, and endothelial cells within hydrogels such as gelatin methacrylate (GelMA), collagen, or hyaluronic acid [57]. These constructs reproduce key features of the pathological synovium in RA. Features include FLS proliferation, cytokine secretion (IL-6, TNF, IL-1β), matrix degrading enzyme production and pannus-like invasion [31]. Incorporation of mechanical loading modules or stiffness-tunable bioinks enables the simulation of joints. This enables assessment of mechanotransduction pathways, major contributors to cartilage erosion in RA [58,59]. Bioprinted osteochondral interface constructs, integrating chondrocytes and osteoblasts across cartilage and bone boundaries, facilitate studies of cartilage-bone crosstalk and osteoclast driven bone resorption [60,61]. These platforms provide powerful ex vivo systems for testing disease-modifying antirheumatic drugs or biologics targeting TNF, IL-6R, or the JAK/STAT axis, while preserving the 3D microarchitecture of human joint tissue [62]. RA is characterized by complex immune infiltration dominated by macrophages, T cells, B cells, and neutrophils. Immune-mimetic bioprinting has enabled the inclusion of these immune subsets in engineered synovial tissues, facilitating the study of cytokine networks and cell-to-cell interactions driving chronic inflammation [63,64,65]. In such constructs, the dynamic interactions between T cells and FLS lead to production of IL-17, IL-21, IFN-γ and GM-CSF, amplifying macrophage activation and ECM remodeling. Controlled embedding of cytokine-releasing microspheres (TNF, IL-1β) or co-printing of T/B cell clusters allows the modeling of lymphoid-like aggregates observed in RA synovium [65]. Bioprinting of vascularized synovial models using endothelial cells and pericytes supports studies of leukocyte adhesion and transmigration across inflamed microvessels [31]. Incorporating osteoclast precursors and bone-matrix scaffolds enables quantification of bone erosion and testing of anti-resorptive agents targeting RANKL and TNF [61]. The coupling of angiogenic and osteolytic pathways within these constructs provides an integrated model of joint destruction and repair. The 3D bioprinting is advancing regenerative strategies for joints damaged by RA by bioprinting of autologous chondrocytes, mesenchymal stromal cells and biodegradable hydrogels that support cartilage regeneration following immune modulation [66]. Smart bioinks incorporating immunoregulatory nanoparticles or controlled-release corticosteroids are under investigation for the local suppression of inflammation and promotion of tissue repair [65]. Moreover, bioprinted joint microtissues can be patient-specific, thus enabling personalized drug testing and precision regenerative therapy, bridging immunopathogenesis and translational medicine in RA.

#### 2.3.2. Type 1 Diabetes (T1D)

Type 1 diabetes (T1D) is a chronic AD characterized by T cell-mediated destruction of pancreatic β-cells, leading to absolute insulin deficiency [67]. Globally, approximately 8.4 million individuals are affected, and the prevalence continues to rise by 2–3% annually [68,69]. The level of the T1D-affected population is projected to reach 13.5–17.4 million by 2040 [68]. T1D is a polygenic disorder, one strongly influenced by the human leukocyte antigen (HLA) region [70,71]. Beyond the HLA region, over 60 non-HLA susceptibility loci have been identified, including CTLA4, PTPN22, IL2RA, INS, KIR and VNTR polymorphisms, which modulate immune tolerance and β-cell antigen presentation [72,73,74]. Environmental triggers interact with this genetic background to initiate β-cell autoimmunity. Enteroviral infections, particularly Coxsackievirus B, have been implicated in disease onset through mechanisms such as molecular mimicry and local β-cell inflammation [75]. Other risk factors, including gut-microbiota dysbiosis, vitamin D deficiency, early-life obesity and diet, contribute to immune dysregulation [76].

The immunopathogenesis of T1D involves a progressive breakdown of central and peripheral immune tolerance, culminating in autoimmune β-cell destruction [77]. Disease initiation likely begins when antigen-presenting cells (APCs) present β cell-derived peptides in pancreatic lymph nodes, activating autoreactive CD4^+^ and CD8^+^ T cells that have escaped thymic negative selection [78]. Activated CD4^+^ T cells support B cell differentiation into plasma cells that secrete anti-β cell autoantibodies, whereas CD8^+^ cytotoxic T lymphocytes directly induce β-cell apoptosis through perforin and granzyme release [79,80]. Within the pancreatic islets, macrophages and dendritic cells amplify inflammation via cytokines such as IL-1β, TNF and IFN-γ, which exacerbate endoplasmic reticulum stress and β-cell death [81,82]. Persistent immune infiltration leads to insulitis, characterized by islet atrophy, reduced pancreatic volume, and loss of insulin secretory function [83]. The interplay between genetic polymorphisms, innate inflammation, and adaptive immune activation results in a self-sustaining autoimmune cascade that defines the clinical course of T1D. The autoimmune destruction of pancreatic β-cells makes this tissue particularly suitable for biofabrication via 3D bioprinting. Several approaches have been developed to reconstruct the islet microenvironment, including the study of immune–endocrine interactions and the development of implantable insulin-producing constructs [67,84]. Recent advances include the bioprinting of human islets within alginate-based bioinks supplemented with decellularized pancreatic ECM [85]. Perrier et al. [86] demonstrated that such constructs retained glucose-responsive insulin secretion for over three weeks. To more closely mimic autoimmune insulitis, future models aim to incorporate patient-derived autoreactive T cells or APCs. This approach could allow direct observation of β cell cytotoxicity and testing of immunomodulatory therapies [87].

Conventional 2D cultures fail to capture the spatial and biochemical complexity of pancreatic islets, which are composed of insulin-secreting β-cells, glucagon producing α-cells and supporting endothelial and stromal components [88]. The 3D bioprinting enables precise spatial deposition of multiple cell types within ECM-mimicking hydrogels, such as alginate, GelMA and collagen, reproducing native architecture and oxygen/nutrient gradients critical for β-cell survival [89]. Bioprinted islet-on-a-chip or vascularized islet organoids recapitulate insulin secretion dynamics in response to glucose stimulation and have been used to investigate cytokine-induced β-cell stress and apoptosis [90]. The immune-mimetic systems were developed with the aim of replicating insulitis and enabling real-time analysis of immune-cell infiltration, β-cell cytotoxicity and cytokine signaling (IL-1β, IFN-γ, TNF) in studies of autoimmune mechanisms in T1D [91]. These systems integrate immune cells, such as autoreactive CD4^+^ and CD8^+^ T lymphocytes, macrophages and dendritic cells into bioprinted pancreatic constructs [29]. The incorporation of microspheres that release inflammatory cytokines within the hydrogel matrix allows chronic activation of immune pathways, thereby mimicking progressive β-cell destruction observed in vivo. These dynamic systems enable evaluating the efficacy of immunomodulatory therapies, including JAK inhibitors, Treg-inducing biologics and encapsulation strategies designed to protect β-cells from immune-mediated damage [92]. A major translational goal of bioprinting T1D models is the fabrication of implantable, vascularized bioartificial islets. Multi-nozzle bioprinters have been employed to co-print β-cell spheroids, endothelial cells and mesenchymal stromal cells in bioinks enriched with ECM peptides, promoting angiogenesis and long-term graft survival. Hybrid constructs combining islet organoids with vascularized scaffolds exhibit improved insulin release and greater hypoxia resistance after transplantation into diabetic rodents [93]. Encapsulation with semipermeable hydrogels (e.g., PEGDA, alginate–chitosan composites) prevents immune rejection while permitting nutrient and insulin diffusion [94]. Integration of patient-derived induced pluripotent stem cells (iPSCs) with high-resolution bioprinting now enables personalized modeling of T1D [95]. Printed constructs derived from iPSC-β cells can recapitulate donor-specific susceptibility to cytokine-mediated apoptosis and serve as screening tools for individualized immunotherapies [96]. Emerging multi-material bioprinting platforms incorporating vascular, immune, and endocrine compartments promise next-generation bio-hybrid pancreas models that could eventually bridge preclinical modeling with cell-based replacement therapy [28]. While challenges remain, bioprinting provides a uniquely modular, tunable system for interrogating disease pathogenesis and developing regenerative solutions for T1D.

#### 2.3.3. Inflammatory Bowel Disease (IBD)

Inflammatory bowel disease (IBD) is a collective term encompassing chronic, relapsing inflammatory disorders of the gastrointestinal (GI) tract, primarily Crohn’s disease (CD) and ulcerative colitis (UC) [97,98]. These conditions are characterized by prolonged mucosal inflammation that leads to structural and functional impairment of the GI barrier. Globally, the incidence and prevalence of IBD are rising across both developed and developing regions, reflecting complex interactions between genetic susceptibility, environmental exposures and immune dysregulation [99].

The pathogenesis of IBD arises from an inappropriate immune response to intestinal microbiota in genetically predisposed individuals [97]. Environmental factors play a pivotal role in modulating gut permeability, altering the microbiome, and initiating mucosal inflammation [100]. Disruption of intestinal homeostasis by smoking, air pollution, high-processed diets, urbanization and antibiotic exposure has been linked to increased IBD susceptibility [101]. Airborne pollutants, including particulate matter (PM_2.5_) and nitrogen oxides, have been associated with disease flares and increased hospitalizations in both CD and UC [102]. These pollutants may promote oxidative stress, epithelial injury and microbial dysbiosis, leading to persistent immune activation. Additionally, global climate change, characterized by temperature extremes, prolonged heatwaves, altered rainfall patterns and droughts, may indirectly influence IBD epidemiology by shifting infectious disease cycles, dietary patterns, and stress-related triggers [103]. Genetically, over 250 loci have been associated with IBD, including variants in NOD2, ATG16L1 and IL23R, which regulate innate immunity and autophagy [104]. The persistent increase in IBD prevalence, coupled with the disease’s chronic relapsing-remitting course, demands a shift toward personalized and preventive management strategies [99]. Understanding environmental determinants and their interaction with host immunity is crucial for developing predictive models of disease activity and optimizing treatment algorithms [103]. Moreover, the integration of bioprinting and organoid technologies is beginning to transform IBD research by enabling human-relevant modeling of mucosal immunity, microbiome interactions and drug responses within engineered intestinal constructs.

3D bioprinting has the potential to enable the creation of human-relevant, multicellular constructs that replicate the intestinal barrier, immune–epithelial interactions and microbial dynamics underlying disease pathogenesis [105,106]. Conventional in vitro models and animal systems inadequately reproduce the spatial organization, biochemical gradients and chronic inflammatory conditions of the human gut. In contrast, 3D bioprinting provides a physiologically tunable environment that permits controlled manipulation of immune, stromal and microbial components, thereby enabling precise analysis of mucosal inflammation, epithelial regeneration and therapeutic response [106,107]. The intestinal epithelium has been bioprinted by layers using patient-derived organoid cells on hydrogel scaffolds, along with underlying fibroblasts and embedded macrophages to model inflammatory crosstalk [108]. These constructs permit assessment of barrier permeability, cytokine secretion and immune cell infiltration. Combining bioprinting with gut-on-chip systems enables dynamic luminal flow, further simulating nutrient absorption and microbial interactions critical in IBD pathogenesis [109]. Bioprinted intestinal models use combinations of epithelial cells, goblet cells, fibroblasts and immune cells embedded within hydrogels such as collagen, GelMA, alginate or Matrigel to reconstruct native-like architecture [110]. Layered constructs featuring crypt-villus topography and perfusable microvascular channels support physiological nutrient flow and epithelial polarity. These systems mimic the tight-junction integrity and mucus-secreting capacity of the intestinal barrier, providing an ideal platform to study the increased permeability and cytokine-driven epithelial disruption observed in IBD [106]. Integration of microbiota or bacterial metabolites within bioprinted systems enables exploration of host–microbe crosstalk. Controlled colonization with commensal or pathogenic strains (e.g., *Escherichia coli*, Bacteroides fragilis) can trigger pattern-recognition receptor activation and downstream inflammatory cascades, reflecting the immune dysregulation characteristic of CD and UC [111]. Such gut-on-chip and bioprinted co-culture systems have been instrumental in modeling dysbiosis-induced epithelial injury and evaluating probiotic or antibiotic interventions. To capture the immunopathological dimension of IBD, immune cells and innate lymphoid cells were co-printed with epithelial layers or introduced via perfusion [105]. These immune-mimetic constructs reproduce proinflammatory cytokine networks (e.g., TNF, IL-1β, IL-6, IL-17A, and IFN-γ) and facilitate study of cell–cell signaling between epithelium, lamina propria and resident immune subsets [112]. Embedding microspheres that release cytokines or bacterial products (e.g., LPS, flagellin) enables the development of chronic stimulation models that mirror long-term mucosal inflammation and fibrosis [14]. Such systems are increasingly used for drug screening and evaluating the responses to anti-TNF, anti-integrin and JAK inhibitors in humanized 3D settings [113]. Multi-material bioprinting further allows incorporation of oxygen gradients, simulating hypoxic niches typical of inflamed gut mucosa and regulating macrophage polarization [114]. Collectively, these immune-integrated constructs provide dynamic, reproducible models of intestinal immunopathogenesis beyond the limitations of 2D or animal models. IBD is marked by repeated cycles of epithelial injury and repair, which can culminate in fibrosis, a pathological accumulation of ECM components and structural remodeling of the intestinal wall. To model these fibrotic processes, bioprinting technologies have been employed to generate fibroblast-enriched lamina propria analogues that recapitulate hallmark features of intestinal fibrosis, including collagen deposition, matrix stiffening and activation of α-SMA^+^ myofibroblasts [115]. These constructs offer a physiologically relevant platform to dissect key signaling pathways, such as TGF-β, IL-13 and the MMP/TIMP axis, that promote fibrogenesis and tissue remodeling [116]. The integration of patient-derived intestinal organoids, immune cells and microbiota samples into bioprinted constructs offers a path toward personalized disease modeling [117]. These individualized IBD-on-a-chip systems are capable of capturing patient-specific immune signatures, barrier defects, and therapeutic responses, thereby supporting the development of precision gastroenterology. Furthermore, progress in vascularized intestinal bioprinting and bioactive scaffolds technologies indicates potential applications in tissue repair or transplantation for severe IBD cases [108]. Standardization of bioink formulations, validation of immune cell functionality and incorporation of biomechanical stimuli will be essential for clinical translation.

#### 2.3.4. Multiple Sclerosis (MS)

Multiple sclerosis is a chronic inflammatory demyelinating disorder of the central nervous system which represents the leading cause of non-traumatic neurological disability in young adults worldwide [118,119,120]. Globally, approximately 2.8 million people live with MS; women are affected about twice as often as men [121]. MS is a complex autoimmune disorder characterized by substantial heterogeneity. The HLA-DR15 haplotype has been identified as a major susceptibility factor [119]. Comprehensive genomic studies have since delineated a broader landscape of MS-related risk, identifying more than 200 non-MHC susceptibility variants: 32 within the MHC region, and one on the X chromosome [120]. These findings underscore the central contribution of immune-regulatory pathways and microglial activation to MS pathogenesis. Environmental factors significantly modulate risk of developing MS. An imbalance in the intestinal microbiota can disrupt immune homeostasis and promote autoreactivity, while Epstein–Barr virus infection has emerged as a near-universal antecedent of MS, conferring a >30-fold increased risk compared to other viral exposures [122,123]. Additional risk factors include obesity, smoking [124,125] and vitamin D deficiency [126]; these are related to inflammation and neurodegenerative cascades induction.

MS pathogenesis arises from a complex interplay between peripheral immune dysregulation and CNS-intrinsic inflammatory processes. Autoreactive T cells are primed in the periphery and migrate across the blood–brain barrier (BBB) via adhesion molecules such as VCAM-1 and ICAM-1, initiating demyelination by targeting oligodendrocyte-derived myelin [127]. These infiltrates amplify inflammation through cytokine cascades, recruiting additional immune subsets and perpetuate tissue injury. Th1 and Th17 cells are principal mediators, secreting proinflammatory cytokines including IFN-γ, TNF, IL-17, and IL-22, while CD8^+^ T cells contribute via FasL-dependent cytotoxicity, driving lesion formation [128,129,130]. Concurrently, B cells act not only as antibody producers but also as antigen-presenting and cytokine-secreting cells, providing a mechanistic rationale for the clinical efficacy of anti-CD20 therapies [131]. Within the CNS, astrocytes and microglia amplify pathology through production of matrix metalloproteinases, reactive oxygen/nitrogen species and TNF, culminating in oligodendrocyte death and axonal degeneration [132]. Collectively, these immune and glial interactions underpin the demyelination, neuroaxonal loss and progressive disability that define MS.

The approach of 3D bioprinting has emerged as a promising technology for modeling the complex neuroinflammatory and demyelinating processes underlying MS. While conventional 2D culture systems and animal models often fail to replicate the cellular diversity, and immune-neural crosstalk, the bioprinted constructs enable the spatially controlled assembly of neural, glial, vascular and immune components within ECM-mimicking hydrogels, thereby allowing precise investigation of cell–cell and cell–matrix interactions under disease-relevant conditions [133,134]. Initial bioprinting studies have successfully demonstrated the biofabrication of neural and glial constructs capable of reproducing the demyelination–remyelination cycle. The use of human-induced pluripotent stem cell-derived neurons, astrocytes and oligodendrocyte precursor cells embedded in GelMA, collagen or fibrin-based hydrogels has enabled the study of myelin sheath formation and axonal regeneration within physiologically relevant mechanical environments [135,136]. These bioprinted neural tissues facilitate assessment of oligodendrocyte maturation and myelination in a vascularized human-relevant model and offer promise as platforms for drug screening [137]. An emerging research direction involves immune-mimetic bioprinting, where constructs are co-printed with microglia, macrophages or lymphocyte subsets to model CNS-specific immune responses [138,139]. Incorporation of inflammatory cues characteristic for MS, such as cytokine-releasing microspheres (e.g., IL-6, IFN-γ, TNF) [140], may allow researchers to perform the controlled simulation of neuroinflammatory niches, enabling analysis of T cell-mediated and macrophage-mediated demyelination. Moreover, adjusting hydrogel crosslinking density and stiffness allows replication of the fibrotic, stiffened milieu characteristic of chronic MS plaques, which is known to influence immune cell activation and glial scar formation [141]. Parallel advances have been made in vascularized and barrier-integrated constructs. Bioprinted BBB models composed of endothelial cells, astrocytes and pericytes recapitulate selective permeability and immune-cell trafficking, providing human-relevant systems for evaluating therapeutic penetration and immune migration across the BBB [142]. When coupled with microfluidic perfusion, these constructs support dynamic modeling of neurovascular inflammation and facilitate high-throughput drug testing [143]. The integration of patient-derived stem cells into these 3D constructs further supports the development of personalized MS models, allowing evaluation of genetic susceptibility and variable therapeutic responses across patients [144]. Although current applications remain largely preclinical, the convergence of neural tissue engineering, immune co-culture and biomaterial innovation positions 3D bioprinting as a transformative tool in MS research, leading toward translation into precision regenerative neurology.

#### 2.3.5. Systemic Lupus Erythematosus (SLE)

Systemic lupus erythematosus (SLE) is a prototypic autoimmune disorder characterized by the production of anti-nuclear autoantibodies (ANA) and deposition of immune complexes in multiple organs, leading to systemic connective tissue inflammation [145,146,147]. It predominantly affects young women, with approximately 90% of cases occurring in females of childbearing age, often resulting in complications that can impact fertility and pregnancy outcomes [148]. Globally, the prevalence of SLE is estimated at 0.3–0.5% and the age-standardized mortality rate remains higher than those of most other AD, accounting for approximately 1.7 to 3.1 in Europe, 1.66 to 2.06 in the USA and 2.1 to 11.1 in Asia [149]. From a genetic perspective, susceptibility to SLE is strongly associated with the HLA class II region, particularly HLA-DRB1, with HLA-DRB1*03:01 linked to the production of anti-Ro and anti-La autoantibodies, and HLA-DR3 correlated with anti-dsDNA antibody formation [150]. High-density single-nucleotide polymorphism analyses have further identified independent risk loci within HLA-DPB1, HLA-G and MSH5, suggesting a complex network of interacting MHC signals [150,151]. Complement pathway deficiencies also confer significant risk due to impairing the clearance of apoptotic debris, thereby perpetuating autoantigen exposure. Additionally, monogenic mutations in DNASE1/DNASE1L3, PRKCD, TREX1, STING and SAMHD1 contribute to aberrantly elevated type I interferon (IFN) levels [150,152]. Environmental triggers also influence disease onset and severity, e.g., viral infection with EBV or cigarette smoking is known to promote disease development [153].

The immunopathogenesis of SLE involves a breakdown of both innate and adaptive immune tolerance. Circulating immune complexes formed by nucleic acids and autoantibodies stimulate plasmacytoid dendritic cells through TLR-mediated pathways, resulting in excessive production of type I interferons, particularly IFN-α [154] This amplifies antigen presentation and promote signaling cascades, enhancing the activation and survival of autoreactive lymphocytes [155]. Active self-antigen presentation predispose CD4^+^ Th cells to secrete proinflammatory mediators (such as TNF, IL-6 and B-cell activating factor), promoting B-cell differentiation and plasma cells production [156]. Plasma cells produce high-affinity autoantibodies that contribute to immune complex formation, driving ongoing tissue damage through complement activation and localized inflammatory responses. Over time, this cycle results in widespread tissue destruction, including renal, cutaneous and vascular damage, establishing the clinical and histopathologic hallmarks of SLE [154].

The approach of 3D bioprinting has significantly enhanced the experimental modeling of SLE, providing human-relevant, multicellular platforms to investigate immune-complex-mediated tissue injury and assess novel therapeutic strategies. While conventional murine models and two-dimensional cell cultures remain foundational for studying SLE immunopathology, they fall short in replicating the structural, cellular, and biochemical complexity of human lupus lesions [145,147]. Bioprinting enables spatially controlled assembly of tissue constructs incorporating immune cells, stromal fibroblasts and vascular elements, effectively mimicking the inflammatory microenvironment seen in SLE-affected tissues such as the kidney, skin and vasculature [157,158]. Using bioinks such as GelMA, collagen and fibrin, researchers have developed perfusable microvascular networks that support immune cell trafficking [8] and deposition of immune complexes, facilitating real-time visualization of complement activation and endothelial damage [158]. These models recapitulate key features of lupus nephritis (LN), including glomerular basement membrane thickening, capillary loop remodeling and immune complex deposition [159]. The incorporation of immune-mimetic components into 3D constructs is crucial. B cells, T cells and monocyte-derived macrophages can be printed into hydrogels, allowing controlled evaluation of autoantibody production, immune complex dynamics and type I interferon signaling [14]. Additionally, embedding cytokine-releasing architectures within hydrogels enables the creation of sustained inflammatory niches that mirror the cytokine-rich microenvironments of SLE tissue. [160] These platforms have proven valuable for investigating key inflammatory pathways, such as JAK-STAT and BTK [161] and for targeted therapy testing. Glomerulus-on-a-chip and kidney-on-bioprint platforms now serve as vital tools for modeling LN. These systems combine podocytes, mesangial cells and glomerular endothelial cells in hydrogel matrices with perfusable channels to simulate immune complex deposition and proteinuria [162]. Bioprinted vascular constructs incorporating endothelial and smooth muscle cells are being used to model lupus-associated vasculopathy and accelerated atherosclerosis [163]. Furthermore, skin models bioprinted with keratinocytes and fibroblasts exposed to IFN-α or UV-B light have replicated cutaneous manifestations of lupus, including photosensitivity and local inflammation, supporting high-throughput drug testing and biomarker identification [164].

#### 2.3.6. Key Immune-Mimetic Applications

**Figure 3:**
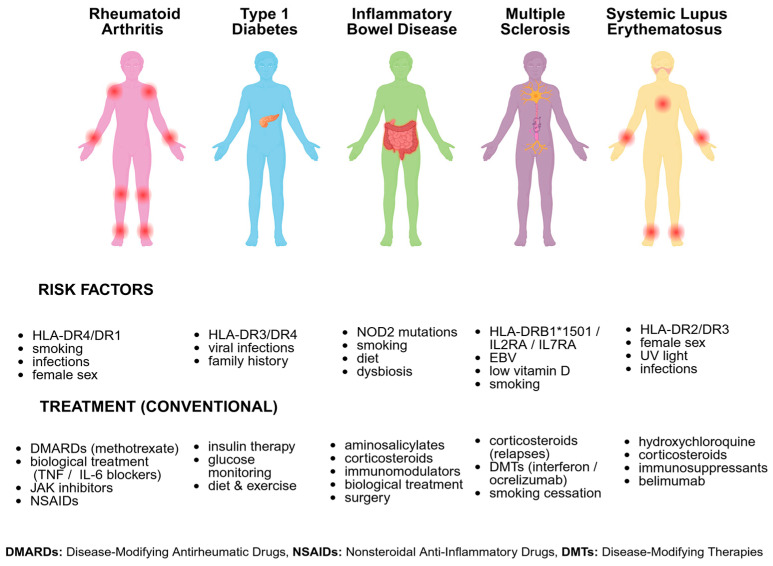
Overview of major AD with their key risk factors and conventional treatments.

A key innovation is the incorporation of immune cells within bioprinted constructs; this includes co-printing strategies such as promoting macrophages to model tissue infiltration and polarization. Another approach is embedding microspheres that release cytokines such as IL-6 or IFN-γ to establish chronic inflammatory niches, sustaining disease-like conditions over the course of weeks [165]. Modulating hydrogel crosslinking enables replication of disease-specific mechanical signatures, thereby influencing immune cell activation and migration [166]. These bioprinted immune-mimetic systems have been applied across representative AD, including RA, T1D, IBD, MS and SLE, capturing their organ-specific pathophysiology (Figure 3). Each condition arises from a combination of genetic predisposition, environmental triggers, infections and hormonal status, while conventional therapeutic management relies on disease-modifying antirheumatic drugs, nonsteroidal anti-inflammatory drugs, corticosteroids, immunomodulators and targeted biological or disease-modifying therapies.

### 2.4. Translational Applications in Drug Screening and Mechanistic Studies

Bioprinted therapeutic constructs for AD are primarily designed to deliver cell-based therapies, bioactive scaffolds or immunomodulatory agents in a controlled and localized manner. (Table 2) The RA pannus model by Lin et al. [31] demonstrated clear responses to methotrexate or TNF inhibitors, validating its use for screening anti-angiogenic or anti-cytokine agents. Such systems could be adapted to test emerging biologics targeting JAK-STAT or IL-17 pathways. In T1D, for instance, subcutaneously implanted constructs containing bioprinted islets within ECM-enriched hydrogels have demonstrated sustained insulin secretion and glycemic responsiveness in preclinical models, offering an alternative to intrahepatic islet transplantation [167]. Furthermore, Bisconti et al. [168] demonstrated that chitosan-based hydrogels provide a biocompatible and degradable platform for synoviocyte encapsulation, offering potential as a local immunomodulatory scaffold for arthritis therapy.

### 2.5. Potential Applications of 3D Bioprinting in Transplantation

The approach of 3D bioprinting is increasingly recognized as a transformative technology in transplantation medicine, providing an avenue to fabricate patient-specific, vascularized, and immune-compatible tissues that may alleviate the global shortage of donor organs [40,174]. Through the layer-by-layer deposition of cell-laden bioinks containing autologous stem cells, extracellular matrix components and bioactive signaling molecules, bioprinting enables the recreation of intricate tissue architectures, such as liver lobules, renal tubules, pancreatic islets and cardiac muscle, that closely mimic their native counterparts [175]. The integration of microvascular and lymphatic networks into printed grafts enhances oxygen and nutrient exchange, a critical step toward achieving long-term graft survival following implantation [40]. Furthermore, combining patient-derived iPSCs or MSCs with immunomodulatory biomaterials could reduce the need for systemic immunosuppression and minimize the risk of rejection [176]. Incorporating immune cells into bioprinted constructs greatly enhances their physiological relevance, providing a more accurate representation of disease-specific immune-tissue interactions. However, this approach remains limited by challenges in sourcing, maintaining and standardizing functional human immune cells. Primary immune cells isolated from patient blood or tissues retain native receptor profiles and authentic cytokine responses, offering valuable patient-specific insight, yet they are hindered by donor-to-donor variability and a short lifespan in culture [29,87]. In contrast, immortalized cell lines such as Jurkat T cells or THP-1 monocytes offer scalability and experimental consistency but only partially replicate in vivo immune activation and antigen presentation [177,178]. In an effort to address these challenges, iPSC-derived immune cells have recently emerged as a promising alternative. Guo et al. [179] demonstrated that iPSC-derived macrophages exhibit functional and transcriptional profiles comparable to primary immune cells, while Ackermann et al. [180] highlighted their potential for disease modeling and drug screening. Together, these advances enable the construction of adaptive immune models, enhancing the precision of personalized studies of disease mechanisms and therapeutic responses.

A particularly promising area of translational progress is 3D bioprinting for corneal transplantation. Corneal blindness affects more than 12 million individuals globally, yet donor tissue availability remains critically limited [181]. Bioprinting enables precise spatial deposition of collagen or GelMA hydrogels seeded with keratocytes and epithelial and endothelial cells, generating transparent and mechanically stable constructs that mimic the native corneal stroma [182]. Recent advances have yielded bioengineered corneas with aligned collagen fibrils and optical properties comparable to native tissue, and which are capable of supporting epithelial cell growth and light transmission [183,184]. Moreover, iPSC-derived corneal epithelial and endothelial cells have been successfully integrated into bioprinted scaffolds, demonstrating early feasibility for customized, immune-compatible corneal grafts [185]. Such approaches could revolutionize ocular transplantation by enabling on-demand, autologous graft fabrication and reducing dependence on allogeneic donors.

Despite such breakthroughs, challenges remain before 3D-printed tissues can enter clinical transplantation pipelines. These include ensuring long-term graft functionality, vascular and neural integration, standardization of bioinks and printing parameters, and establishing regulatory frameworks for safety and quality control [186]. As the field matures, 3D bioprinting is poised to become a promising tool for next-generation regenerative transplantation, integrating engineering precision with personalized medicine.

### 2.6. Legal Regulation of Bioprinted Products

Safety considerations encompass biocompatibility, sterility, immunogenicity and containment, especially when using gene-edited or patient-derived cells [187]. According to the Food and Drug Administration, FDA (USA), in accordance with section 212 of the Small Business Regulatory Enforcement Fairness Act (Public Law 104-121), the key regulations for establishments that manufacture human cells, tissues or cellular or tissue-based products (HCT/Ps) are as follows: Title 21 of Code Federal Regulations part 1270 and 1271; Guidance on Regenerative Medicine Advanced Therapies (RMAT) Designation (Section 3033 of the 21st Century Cures Act). According to the European Medical Administration, EMA (EU), the following regulations are mandatory: regulation EC No 1394/2007 for use of Advanced Therapy Medicinal products (ATMP); EU No 2017/745 for Medical Device Regulation.

## 3. Limitations and Future Directions

Although bioprinting strategies remain largely experimental, their integration into personalized medicine is progressing steadily. Advances in preclinical modeling and automation and the early clinical studies of engineered tissues are driving this progress [188]. Notably, the most mature applications of 3D bioprinting have emerged within oncology, where multicellular tumor models allow the reconstruction of complex tumor microenvironments and immune interactions [189,190]. These advances in cancer bioprinting underscore the translational potential of 3D bioprinting for modeling autoimmune diseases, where similar immune–stromal signaling dynamics govern tissue injury and repair. However, substantial scientific, technical, and regulatory challenges must be addressed before widespread adoption in clinical or drug discovery settings. Most current models do not fully capture the complexity of human immune architecture, and often lack the organized lymphoid tissue and sustained antigen-presenting capabilities necessary for modeling the flare-remission cycles of AD [191]. Consequently, chronic immune activation and tolerance mechanisms are incompletely represented. Another significant challenge is the absence of functional vascularization in most bioprinted systems, which restricts nutrient and oxygen diffusion, impairs metabolic activity and limits long-term cell survival. Efforts to incorporate endothelial-lined microchannels and microfluidic perfusion systems are underway to better mimic the physiological flow and enhance graft viability [192]. The generation of patient-specific cell types, particularly immune and stromal components, is complicated by variability in donor cell yield, differentiation efficiency, and printing protocols hindering standardization and reproducibility. Regulatory challenges also persist, as there is no globally harmonized framework for bioprinted constructs, and definitions for print fidelity, cell viability, immunogenicity and bioink quality differ across jurisdictions. Natural or decellularized bioinks present additional concerns regarding material traceability, batch variability and potential immune reactivity [192]. Another major challenge is the lack of standardization. Differences in printer calibration, bioink formulation and cell-handling protocols often result in inconsistent construct geometry, mechanics and biological performance [134]. Moving forward, establishing shared benchmarks, standardized reporting practices and cross-laboratory validation will be essential to improving reproducibility and supporting the safe, reliable translation of immune-integrated bioprinted models into clinical use [193]. Future models are anticipated to integrate bioprinting with AI-driven multiomics platforms to simulate blood and lymphatic flow, enabling precise analysis of immune cell trafficking and tissue infiltration, enhancing the accuracy of immunopathology and therapeutic prediction through dynamic systems-level analyses [194,195].

## 4. Conclusions

The area of 3D bioprinting is rapidly establishing itself as a transformative tool in AD modeling, bridging the long-standing gap between conventional 2D cultures and animal models. Its unique ability to replicate complex immune-tissue interactions within precisely controlled, patient-specific architectures provides an unprecedented platform for dissecting disease mechanisms and testing novel therapeutics. As biomaterials continue to evolve, particularly in their tunability, biofunctionality, and compatibility with immune cell co-cultures, these systems are becoming increasingly reflective of human physiology. However, key challenges remain. Accurately representing immune system complexity, achieving stable vascularization and establishing standardized printing and validation protocols are all essential steps toward broader adoption. Moreover, the absence of unified regulatory and manufacturing frameworks continues to hinder clinical translation. Addressing these challenges will be critical to realizing the full potential of 3D bioprinting as a translational platform, one capable of transforming both mechanistic research and personalized medicine for autoimmune diseases over the coming decade.

## Figures and Tables

**Table 1:** Comparative overview of 3D bioprinting techniques for AD modeling.

Technique	Typical Resolution (µm)	Cell Viability (%)	Key Advantages	Main Limitations	Representative Applications in AD Modeling	Reference/Year
Extrusion-based bioprinting	100–300	~75–90	Supports highly viscous bioinks (e.g., alginate/methylcellulose); enables macroporous scaffolds with controlled architecture; maintains islet morphology and glucose responsiveness	Potential shear stress during printing; reduced insulin response over time; limited diffusion in larger constructs	Pancreatic islet constructs for T1D (viable, glucose-responsive islets within Alg/MC scaffolds)	Duin et al., 2019 [30]
	100–300	70–90	Handles viscous, high-cell-density bioinks; supports multi-material fabrication; simple and cost-effective setup	High shear stress on cells; limited resolution; slower printing speed	Synovial and osteochondral constructs for RA (3D co-culture pannus tissue models)	Lin et al., 2021 [31]
Inkjet bioprinting	~20–30	~87–90	High printing resolution and throughput; non-contact cell placement; compatible with low-viscosity bioinks; minimal nozzle clogging with PEG-based hydrogels	Limited to low-viscosity inks; potential for cell damage due to thermal stress; low mechanical strength of printed hydrogels	Cytokine gradient mapping and cell–matrix interaction studies in inflammatory and musculoskeletal models (RA, IBD)	Gao et al., 2015 [32]
Laser-assisted bioprinting	10–50	85–95	High precision and resolution; supports delicate cell types like stem cells; enables micro-structured tissues (e.g., cornea, retina) with minimal shear stress	High operational cost; complex setup and calibration; limited scalability for large constructs	Bioengineered corneal and retinal constructs for studying immune privilege, inflammation, and tissue repair mechanisms in ocular autoimmune diseases (e.g., uveitis, autoimmune keratitis)	Kim et al., 2025 [33]
3D-embedded/SWIFT bioprinting	100–400	>90	Enables embedded vascular networks within densely cellular organoid matrices; supports perfusion and long-term viability; maintains native tissue microarchitecture; scalable to organ-level constructs	Limited resolution below 400 µm due to organoid size; complex fabrication workflow; slow printing and perfusion setup; incomplete endothelialization of channels	Perfusable, immune-vascularized microtissues for modeling inflammation, hypoxia, and tissue–immune cell interactions in diseases such as RA, SLE, or T1D	Skylar-Scott et al., 2019 [34]

**Table 2:** Overview of 3D bioprinting strategies in AD models.

Disease Model	Cells Used	Bioink/Scaffold	Key Features & Innovations	Reference/Year	Main Application
Rheumatoid Arthritis	MH7A synoviocytes, EA.hy926 endothelial cells	Gelatin/alginate hydrogel	TNF induced VEGF/ANG expression, methotrexate response	Lin et al., 2021 [31]	Tissue modeling, drug screening
Rheumatoid Arthritis	Human fibroblast-like synoviocytes	Chitosan–Matrigel hydrogel composite	Stable 3D FLS culture mimicking synovial microarchitecture	Bisconti et al., 2024 [168]	Synovial tissue modelling, drug testing
Rheumatoid Arthritis (joint anatomy)	(imaging data)	PLA or photopolymer resin (no cells)	CT-based RA joint 3D prints for anatomical erosion	Kleyer et al., 2017 [169]	Surgical education, visualization
Type 1 Diabetes	INS1E β-cell line, rat and human pancreatic islets	1.5% ultrapure alginate hydrogel	Preserved islet viability and glucose-stimulated insulin secretion	Hermanns et al., 2025 [170]	Islet transplantation optimization and metabolic modeling
Type 1 Diabetes	Primary human islets, iPSC-derived islets	Alginate/methylcellulose bioink (3%/6%)	Human and iPSC-derived islets; maintained glucose responsiveness and gene expression	Poklar et al., 2025 [171]	Personalized islet bioprinting, autologous cell therapy
Inflammatory Bowel Disease	RAW 264.7 macrophages; in vivo Balb/c mouse model	Silk fibroin/alginate/hyaluronic acid hydrogel with mesalazine + chitosan:TNF-α siRNA	Hydrogels reduced inflammation and improved mucosal healing in vivo	Yıldız et al., 2025 [172]	Oral bioprinted hydrogel for combinatorial IBD therapy
Inflammatory Bowel Disease	Patient-derived MSCs, fibroblasts, endothelial and epithelial cells	GelMA hydrogel	Promotion of epithelial repair, tight junction formation, fibroblast chemotaxis, and angiogenesis	Perini et. al, 2025 [173]	Regenerative medicine platform for IBD and mucosal healing

## Data Availability

No new data were created or analyzed in this study. Data sharing is not applicable to this article.
